# Simple Analysis of Lipid Inhibition Activity on an Adipocyte Micro-Cell Pattern Chip

**DOI:** 10.3390/biom8020037

**Published:** 2018-06-04

**Authors:** Gi Yong Kim, Su-Jin Yeom, Sung-Chan Jang, Chang-Soo Lee, Changhyun Roh, Heon-Ho Jeong

**Affiliations:** 1 Biotechnology Research Division, Advanced Radiation Technology Institute, Korea Atomic Energy Research Institute, 29 Geumgu-gil, Jeongeup-si, Jeonbuk-do 56212, Korea; kgyong@kaeri.re.kr (G.Y.K.); scjang@cpri.re.kr (S.-C.J.); 2 Department of Chemical Engineering, Chungnam National Univiersity, 99 Daehak-ro, Yuseong-gu, Daejeon 34134, Korea; shiner930@gmail.com (S.-J.Y.); rhadum@cnu.ac.kr (C.-S.L.); 3 Department of Chemical and Biomolecular Engineering, Chonnam National University, 50, Daehak-ro, Yeosu-si, Jeollanam-do 59626, Korea

**Keywords:** micro-contacting, orlistat, quercetin, 3T3-L1 cell, anti-obesity agents

## Abstract

Polydimethyl-siloxane (PDMS) is often applied to fabricate cell chips. In this study, we fabricated an adipocyte microcell pattern chips using PDMS to analyze the inhibition activity of lipid droplets in mouse embryo fibroblast cells (3T3-L1) with anti-obesity agents. To form the PDMS based micropattern, we applied the micro-contact printing technique using PDMS micro-stamps that had been fabricated by conventional soft lithography. This PDMS micro-pattern enabled the selective growth of 3T3-L1 cells onto the specific region by preventing cell adhesion on the PDMS region. It then allowed growth of the 3T3-L1 cells in the chip for 10 days and confirmed that lipid droplets were formed in the 3T3-L1 cells. After treatment of orlistat and quercetin were treated in an adipocyte micro-cell pattern chip with 3T3-L1 cells for six days, we found that orlistat and quercetin exhibited fat inhibition capacities of 19.3% and 24.4% from 0.2 μM of lipid droplets in 3T3-L1 cells. In addition, we conducted a direct quantitative analysis of 3T3-L1 cell differentiation using Oil Red O staining. In conclusion, PDMS-based adipocyte micro-cell pattern chips may contribute to the development of novel bioactive compounds.

## 1. Introduction

Obesity, which arises from an imbalance of energy intake and expenditure, has a tremendous socioeconomic impact at an epidemic level. The World Health Organization (WHO) reported that two billion people suffer from obesity [1,2]. Obesity causes numerous complications such as hypertension, gallbladder disease, cardiovascular disease, heart failure, coronary artery disease, and stroke [3,4]. Although many medications have been used to manage obesity for many years, the global obese population has doubled in the past two decades. Thus, significant research on development of a lipid inhibitor for obesity is required. At present, only orlistat [(*S*)-((*S*)-1-((2*S*,3*S*)-3-hexyl-4-oxooxetan-2-yl)tridecan-2-yl) 2-formamido-4-methylpentanoate], an anti-obesity agent, which is commonly used as an anti-obesity medication, has been approved by the Food and Drug Administration (FDA) [5,6]. Although orlistat has shown efficacy in reducing obesity, there have been adverse side-effects [7,8]. Hence, screening for novel bioactive compounds is important to prevent or reduce the risks of various obesity complications. Quercetin, [2-(3,4-dihydroxyphenyl)-3,5,7-trihydroxy-4*H*-chromen-4-one], another drug candidate, is a plant polyphenol from the flavonoid group, found in natural sources, such as fruits, vegetables, and grains [9]. Quercetin has been reported to activate the inhibition of the oxidation of low-density lipoproteins in vitro, anti-obesity agents, free radical scavengers, protein kinase enzyme inhibitors, and estrogen receptors [10,11,12]. The demand for a biological screening assay platform has been issued to efficiently analyze these kind of drug candidates.

Micro-chip methods are useful analytical tools for researching the cell viability of biomolecules such as proteins, and DNA, because of its advantages, such as easy control and fabrication, biocompatibility and high sensitivity [13,14,15,16]. Polydimethyl-siloxane (PDMS) can be easily and inexpensively fabricated by replicating the polymer against a mold with micro-patterns. Polydimethyl-siloxane has also been utilized in a rapid prototyping process designed for microsystems employing micro-contact printing and microchip devices [17,18]. Here, we demonstrate a simple analysis of inhibition activity of lipid droplets on an adipocyte micro-cell pattern chip using PDMS micro-stamps through micro-contacting to allow rapid and efficient cell patterning of mouse embryo fibroblast cells (3T3-L1). The fabricated PDMS microstructures act as spatial and biological barriers that cause cell adhesion in the cell region. In an adipocyte micro-cell pattern chip, 3T3-L1 cells are successfully cultured and differentiated at low cost and using a small quantity of reagents. Also, orlistat and quercetin anti-obesity agents show inhibition of lipid droplets in 3T3-L1 cells from the designed adipocyte micro-cell pattern chip.

## 2. Materials and Methods

### 2.1. Materials

SU-8 photo-resists were purchased from Microchem (Newton, MA, USA); the 0.45 μm syringe filter and Sylgard 184 PDMS prepolymer were purchased from Dow Corning (Midland, MI, USA); and the Dulbecco’s modified Eagle’s medium (DMEM), fetal bovine serum (FBS), penicillin-streptomycin (PS), and 0.25% trypsin-ethylenediaminetetraacetic acid (EDTA) were purchased from Gibco (New York, NY, USA). Oil red O was purchased from Cayman Chemical (Adipogenesis Assay kit, Ann Arbor, MI, USA). Orlistat and quercetin were purchased from Sigma-Aldrich (St. Louis, MO, USA). We used 3T3-L1cells provided by American Type Culture Collection (ATCC, Manassas, VA, USA). All other chemicals used were of analytical or research grade.

### 2.2. Characterization

All optical images were acquired with a microscope (Nikon, TE-2000, Tokyo, Japan) equipped with a high-resolution charged couple device (CCD) camera (CoolSnap, Roper Science, Tucson, AZ, USA). 3T3-L1 cell dyeing images were taken using a Leica microscope (Leica DFC500 R2 Digital camera & SW kit, Wetzlar, Germany). The cell diameter was measured using Image-pro plus (Media cybernetics, Carlsbad, CA, USA). The absorbance was measured using a microplate reader (Infinite^®^, Tecan Co, ‎Zürich, Switzerland).

### 2.3. Fabrication of the Adipocyte Micro-Cell Pattern Chip

The adipocyte micro-cell pattern chip was fabricated using conventional soft lithography. Polydimethyl-siloxane micro-stamps were fabricated using PDMS prepolymer and a curing agent. In the detailed method described, silicon masters were fabricated using an SU-8 photoresist through a photolithography method. A PDMS prepolymer and a curing agent were mixed at a 10:1 (*w/w*) ratio and inserted into a vacuum oven to remove the air bubbles. The PDMS and curing agent were poured onto the silicon master and cured in a 65 °C oven for 12 h [16]. The cured PDMS micro-stamp was then peeled from the silicon master. Each PDMS micro-stamp was cut such that it formed an open-ended network. To make the thin layers of the PDMS solution, the 3:1 (*w/w*) ratio of PDMS prepolymer and a curing agent were dissolved in chloroform (160 mg/mL). The PDMS solution was poured onto a glass substrate and spin-coated at 1000 rpm for 20 s. The chloroform was evaporated during spin-coating. The PDMS micro-stamp was put over the spin coated PDMS solution with a thin layer using micro-contact printing. The PDMS micro-stamp with PDMS solution was quickly transferred to the cell culture plate and then underwent pre-curing in a 65 °C oven for 20 min. After peeling off the PDMS micro-stamp, the adipocyte micro-cell pattern chip was kept at 65 °C for 6 h.

### 2.4. Cell Culture and Patterning

**Figure 1:**
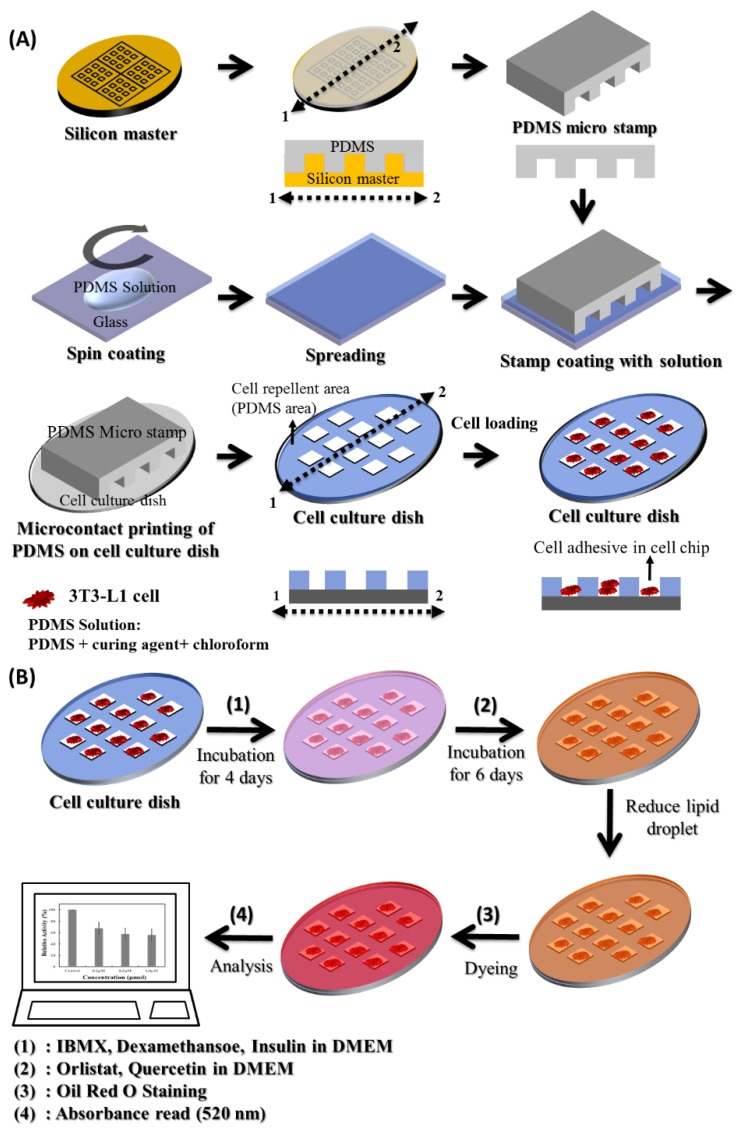
Schematic diagram showing an adipocyte micro-cell pattern chip. (**A**) Fabrication of the micro-cell pattern chip. PDMS: polydimethyl-siloxane. (**B**) Schematic of mouse embryo fibroblast cell (3T3-L1) differentiation by anti-obesity agents and 3T3-L1 cell differentiation analyzed by Oil Red O staining. IBMX: 3-isobutyl-1-methylxanthine; DMEM: Dulbecco’s modified Eagle’s medium.

In this study, 3T3-L1 cells were cultured in a cell culture dish using a medium at 37 °C in a humidified atmosphere with 5% CO_2_. The medium of grown 3T3-L1 cells was removed from the cell culture dish, which was washed three times using phosphate buffered saline (PBS) (pH 7.2). The cells were suspended using 0.25% trypsin-EDTA and centrifuged at 1000 rpm for 1 min. After centrifugation, the 3T3-L1 cells were injected (20 μL) into the micro-cell pattern chips (1.1 cm × 1.1 cm) with a concentration of 5 × 10^4^ cells in the medium. The unbound cells were washed with a fresh medium. The medium was comprised of DMEM, FBS and PS at 89%, 10%, and 1%, respectively (Figure 1A).

### 2.5. 3T3-L1 Cell Differentiation on an Adipocyte Micro-Cell Pattern Chip and Effect of Anti-Obesity Agents Using Oil Red O Protocol

Mouse embryo fibroblast (3T3-L1) cells were cultured on an adipocyte micro-cell pattern chip. To promote 3T3-L1 cell differentiation, confluent 3T3-L1 preadipocytes were stimulated for 4 days with an inducer in a medium (1 μM dexamethasone, 10 μg/mL insulin and 3-isobutyl-1-methylxanthine (IBMX) 0.5 mM) [19,20]. Next, they were maintained in a medium supplemented with orlistat or quercetin at a concentration of 0.2 or 0.5 μM, respectively, for 6 days. To observe the effects of orlistat and quercetin on the inhibition activity of lipid droplets, orlistat and quercetin were treated every 2 days for 10 days. To analyze the inhibition activity of the lipid droplets from anti-obesity agents, Oil Red O staining was used. Oil Red O is a dye that strongly stains lipids. First, the Oil Red O solution was prepared by diluting it in distilled water (3:2) and was then passed through a 0.45 μm syringe filter (working solution). The working solution was prepared freshly for the experiment and filtered once, immediately before use. Second, the medium was removed from the adipocyte micro-cell pattern chip and washed once with PBS. Third, the 3T3-L1 cells were fixed with 10% formalin in PBS for 1 h at room temperature. The formalin was completely washed using isopropanol and dried. Finally, the Oil Red O working solution was combined with the adipocyte micro-cell pattern chip for 2 h at room temperature, washed with distilled water, and dried. In addition, to eluting the dye, 100 μL of 100% isopropanol was poured into each adipocyte micro-cell pattern chip for 10 min at room temperature on an orbital shaker, and the Oil Red O was completely dissolved in isopropanol. The absorption was measured at 520 nm using a micro plate reader (Figure 1B) [21,22].

### 2.6. Statistical Analysis

All values are presented as means and standard deviations (SD). Differences in the data between groups were compared by one-way analysis of variance (ANOVA), using Student’s *t* tests. Correlations were assessed using a Person’s correlation coefficient (PCC). A *p*-value of < 0.05 was considered significant.

## 3. Results and Discussion

### 3.1. 3T3-L1 Cell Growth on an Adipocyte Micro-Cell Pattern Chip

Figure 1 shows the overall schematic diagram of the simple fabrication of the adipocyte micro-cell pattern chip with two distinctive regions, the PDMS region and the cell adhesion region (Figure 1A). The adipocyte micro-cell pattern chip successfully enabled the selective growth of 3T3-L1 cells onto the cell adhesion region. The cell membrane surface consists of an extracellular matrix (ECM) of proteins (e.g., fibronectin, collagen, selectin, integrin) to increase the cell attachment through simple adsorption. Although these cell surface biomolecules have hydrophilic characteristics, PDMS polymers have a hydrophobic characteristic, resulting in difficulty attaching onto the PDMS area of adipocyte micro-cell pattern chips. Mouse embryo fibroblast (3T3-L1) cells were successfully attached to the adhesion region of the adipocyte micro-cell pattern chip. This adipocyte cell pattern chip provided 49 patterns on the single micro-stamp. The adipocyte micro-cell pattern chip allowed 3T3-L1 cell growth on the chip for 10 days. In addition, we performed a direct quantitative analysis of 3T3-L1 cell differentiation using Oil Red O staining (Figure 1B). Therefore, this system provided a simple and efficient method of fabrication of a cell pattern chip and selective 3T3-L1 cell growth on an adipocyte micro-pattern chip.

### 3.2. 3T3-L1 Cell Proliferation by Pattern Size of Cell Pattern Chip

**Figure 2:**
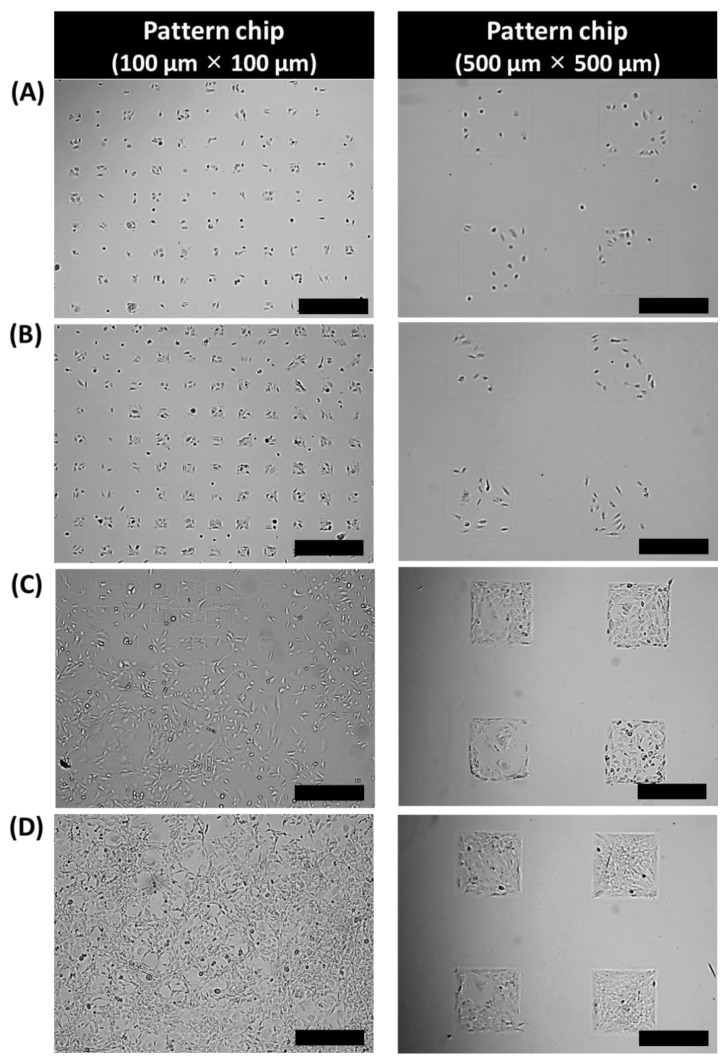
Optical images of 3T3-L1 cells on the adipocyte micro-cell pattern chip after (**A**) 2 h, (**B**) 12 h, (**C**) 24 h, and (**D**) 72 h. Scale bar indicates (left panel) 100 μm and (right panel) 300 μm.

Figure 2 shows optical images of 3T3-L1 cells in different sized micro-cell pattern chips. In a previous study, various live cells (e.g., HeLa, HepG2, MCF-7) were specifically patterned on the culture plates (100 μm × 100 μm) [14]. However, after day one, a 100 μm × 100 μm adipocyte micro-cell pattern chip was shown to have spatial limitations during 3T3-L1 cell proliferation (Figure 2C,D). Mouse embryo fibroblast (3T3-L1) cells are bigger than other cells (average mature 3T3-L1 cell stature ≥14.9 μm) [23]. Therefore, in this study, 3T3-L1 cell differentiation was considered to make a 500 μm × 500 μm adipocyte micro-cell pattern chip. After three days of 3T3-L1 cell seeding, cell proliferation was strictly limited to the cell adhesive area (500 μm × 500 μm).

### 3.3. 3T3-L1 Cell Differentiation on an Adipocyte Micro-Cell Pattern Chip

**Figure 3:**
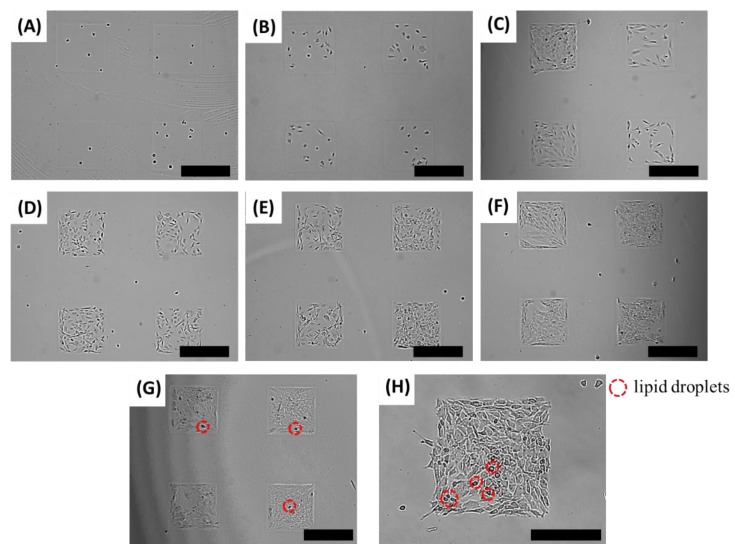
Optical images of the adipocyte micro-cell pattern chip after being cultured for (**A**) 0.5 h, (**B**) 12 h, (**C**) 1 day, (**D**) 2 days, (**E**) 3 days, (**F**) 4 days, (**G**) 6 days and (**H**) 8 days with 3T3-L1 cells on an adipocyte micro-cell pattern chip. Scale bar indicates (**A**–**G**) 500 μm and (**H**) 300 μm.

Figure 3 shows the adipocyte differentiation on an adipocyte micro-cell pattern chip at different times (1 h, 12 h, 1 day, 2 days, 3 days, 4 days, 6 days and 8 days). We found that the lipid droplets in the 3T3-L1 cells did not appear for four days (Figure 3A–F). It was confirmed that lipid droplets were formed in 3T3-L1 cells two days after adding the adipocyte differentiation inducer in the medium (Figure 3G). After eight days of incubation, we successfully observed that an adipocyte micro-cell pattern chip had enabled lipid droplets in the 3T3-L1 cells (Figure 3H)

### 3.4. Inhibition Activity of Anti-Obesity Agents on an Adipocyte Micro-Cell Pattern Chip

**Figure 4:**
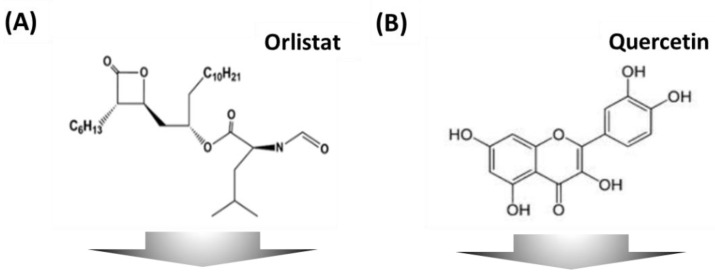

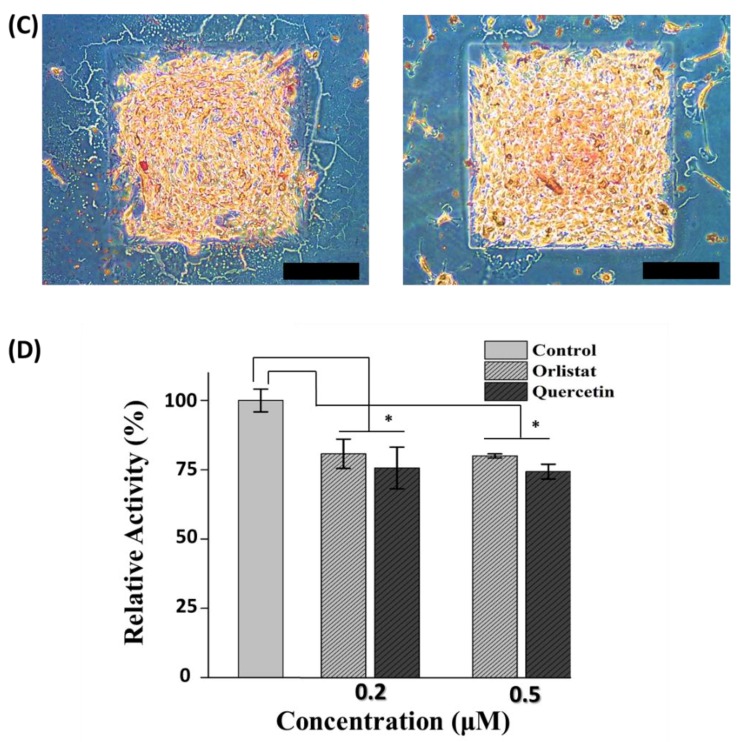
Chemical structure of (**A**) orlistat and (**B**) quercetin. (**C**) Optical images of 3T3-L1 cells were cultured for six days with anti-obesity agents stained with Oil Red O. (**D**) 3T3-L1 cell differentiation anti-obesity activity from orlistat and quercetin. Scale bar indicates 200 μm. Results were analyzed by a one-way analysis of variance (ANOVA) test (*p < 0.001 control vs., orlistat and quercetin group).

As shown in Figure 4, the lipid droplets in 3T3-L1 cells were stained by Oil Red O to determine the inhibition activity of anti-obesity agents. The chemical structures of orlistat and quercetin are presented in Figure 4A,B. Figure 4C shows the formation of lipid droplets in 3T3-L1 cells indicated by red dye on the adipocyte micro-cell pattern chip. It was clearly shown that the hydrophobic PDMS region is able to selectively grow 3T3-L1 cells and enable lipid droplets in differentiated 3T3-L1 cells in the Oil Red O analysis. Therefore, we demonstrated that an adipocyte micro-cell pattern chip allows cell growth and differentiation. In addition, we found that quercetin exhibited a fat inhibitory capacity of 24.4% (*p* = 0.0012), 26.2% (*p* = 0.0009) from 0.5 μm of lipid droplets in 3T3-L1 cells, indicating anti-obesity activity (*p* = control vs. orlistat and quercetin groups), as shown in Figure 4D.

## 4. Conclusions

In summary, we demonstrated a screening system based on inexpensive and facile platform technology using an adipocyte micro-cell pattern chip system in a simple process. Our approach enables systematic analysis of the inhibition activity of lipid droplets in 3T3-L1 cells. The simple adipocyte micro-cell pattern chip allowed consistent 3T3-L1 cells attachment, well-confined cell spreading, and proliferation with high efficiency and reliability. Furthermore, in this study, proof of the principle demonstration was obtained by direct quantitative analysis of 3T3-L1 cell differentiation using Oil Red O staining for lipid droplets in an adipocyte micro-cell pattern chip. We showed that quercetin has a higher anti-obesity activity than orlistat (maximum 5.7%) from its reduced adsorption intensity at 520 nm an adipocyte micro-cell pattern chip. In addition, the cell pattern chip technology used in this study is able to fabricate cell chip of various sizes and shaped according to the cell type. Although, it has several limitations, such as being a costly and complicated process compared to two-dimensional (2D) cell culture, we believe that our method can be readily employed for the screening of anti-obesity agents to reduce the obesity rate. Thus, micro-cell chip technology might be expanded to discover a variety of bio-compounds on cell pattern chips.
